# Tumor-associated macrophage-based predictive and prognostic model for hepatocellular carcinoma

**DOI:** 10.1371/journal.pone.0325120

**Published:** 2025-07-02

**Authors:** Changquan Shang, Tiancong He, Yi Zhang

**Affiliations:** Department of Surgical Oncology, Minhang Branch, Fudan University Shanghai Cancer Center, Shanghai, China; Xiangya Hospital Central South University, CHINA

## Abstract

Hepatocellular carcinoma (HCC) is a prevalent malignancy influenced by the interplay between the immune system and tumor progression, but the detailed biological mechanism still elusive. To address this, we integrate single-cell RNA sequencing (scRNAseq) data with bulk sequencing data to investigate the prognostic significance of tumor-associated macrophages (TAMs) signatures in HCC. Utilizing bioinformatics approaches, including differential gene expression analysis, Cox regression, and logistic regression modeling, we constructed a robust prognostic model that effectively stratifies HCC patients into distinct risk groups with significant differences in survival outcomes. Applying our model to multiple HCC cohorts, robust predictive and prognostic performances were observed. Moreover, examination of the tumor microenvironment (TME) revealed distinct patterns of immune cell infiltration between high-risk and low-risk patient groups, which may contribute to the poorer outcomes observed in high-risk patients. Finally, drug sensitivity and AutoDock simulations suggest that the signature genes we identified could be potential targets for HCC therapy. In summary, this study provides novel insights into the HCC tumor microenvironment and its interaction with TAMs, offering a prognostic model with potential for improving patient stratification and guiding the development of novel therapeutic approaches. Future research ought to concentrate on confirming our findings in larger, prospective studies and examining the functional implications of TAMs in HCC progression.

## Introduction

Liver cancer is the 6th largest primary cancer site and the 4th largest cause of cancer deaths. Hepatocellular carcinoma (HCC), which makes up about 90% of liver cancers, is a prevalent malignancy whose progression is significantly shaped by immune system interactions [1]. HCC typically arises in the context of chronic inflammatory liver disease and is strongly associated with risk factors including HBV, HCV, heavy alcohol consumption, and conditions like obesity, type 2 diabetes, and non-alcoholic fatty liver disease (NAFLD). These factors contribute to liver damage and increase the likelihood of HCC development. Curative treatments, including surgical excision, liver transplant, and radiofrequency ablation, can be used in early-stage disease. However, most patients present with advanced HCC, for which systemic therapies, such as tyrosine kinase inhibitors and immune checkpoint inhibitors, offer only limited survival benefits. Despite advances in therapeutic strategies, the prognosis of patients with advanced HCC remains dismal.

In recent years, substantial efforts have been devoted to identifying reliable prognostic biomarkers to predict the HCC clinical outcomes and to guide personalized treatment strategies. Prognostic biomarkers for HCC encompass a wide range of molecular features, including genetic mutations, gene expression signatures, and epigenetic alterations. For example, TERT promoter mutations, one of the most frequent genetic mutations in HCC, have been strongly associated with malignant transformation, poorer prognosis, and a heightened risk of recurrence [2]. Kornelius Schulze and his colleagues indicated that TP53 mutations are another common marker of HCC, whose mutational status are often linked to adverse outcomes and uncontrolled growth regulation of tumor cells [3]. Additionally, the over-expression of immune checkpoint genes, such as PD-L1, CTLA-4, LAG3, and TIM3, has been frequently correlated with unfavorable prognosis in HCC patients, with high levels of PD-L1 expression particularly indicative of a diminished response to immunotherapy [4]. Overexpression of glypican-3 (GPC3) has also emerged as a hallmark of aggressive tumor phenotypes, correlating with shortened overall and disease-free survival, and representing a promising therapeutic target in HCC [5]. Recent studies have also identified PIAS family genes as prognostic indicators, whose expression levels are associated with immune modulation and sensitivity to chemotherapeutic agents in HCC [6]. In the context of epigenetic alterations, DNA hypermethylation of tumor suppressor genes (e.g., GSTP1, RASSF1A, and SOCS1), have been widely reported in HCC tissues and are associated with more aggressive tumor behavior and lower survival rates [7]. In parallel, the downregulation of microRNAs (miRNAs), such as miR-122 and miR-26, has been implicated in promoting tumor progression and poor patient survival [8]. Moreover, Ma et al. demonstrated that IGF2 BP3 promotes HCC progression by modulating the phenotypes of macrophages and CD8 ⁺ T cells within the tumor microenvironment, further illustrating the interplay between oncogenic signaling and immune regulation in HCC [9]. Despite these advances, the predictive accuracy and clinical applicability of these biomarkers remain suboptimal, highlighting the need for more integrative and mechanistically informed approaches to improve prognostication and guide therapy selection in HCC.

Increasing studies demonstrated that tumor immune microenvironment (TIME), comprising tumor, immune, and inflammatory cells, plays a pivotal role in HCC pathogenesis [10,11]. Among them, tumor associated macrophages (TAMs) are central regulators of tumor progression, angiogenesis, and immune suppression. TAMs exhibit remarkable functional heterogeneity, and can be sculpted into divergent functional phenotypes, M1 and M2, each with its own distinct attributes and functions. M1 TAMs are linked to antitumor immunity, unleashing inflammatory cytokines and reactive oxygen species that are cytotoxic to tumor cells. In stark contrast, M2 TAMs foster tumor growth and progression by secreting growth factors, cytokines, and chemokines that suppress the immune system and promote angiogenesis and tissue remodeling. In HCC, the TIME is often skewed toward the M2 phenotype, facilitating immune evasion and tumor progression. Thus, unraveling the mechanisms that govern TAM polarization and function is essential for devising effective therapeutic strategies to reprogram these cells, thereby enhancing anti-tumor immunity and improving patient outcomes. Additionally, recent investigations have underscored the potential of TAMs as prognostic biomarkers in HCC [12]. Their abundance and polarization state within the tumor microenvironment have been shown to correlate with clinical outcomes, such as tumor stage, recurrence rates, and patient overall survival. Consequently, the exploration of TAMs in HCC is indispensable for comprehending their impact on cancer development, immune suppression, and for identifying potential therapeutic targets and prognostic biomarkers. By targeting and modulating TAMs, it may be able to enhance anti-tumor immunity, improve therapeutic outcomes, and ultimately prolong HCC patients’ survival.

In this study, we integrated scRNA-seq data with bulk sequencing data (TCGA-LIHC) to identify TAM-associated genes for predicting HCC patient’s prognosis. Using these genes as inputs, we developed a prognostic prediction model for HCC patients. When applied to independent external validation cohorts, the model demonstrated robust prognostic and predictive performance. Moreover, notable disparities in TME and drug susceptibility were also noted between the low-risk and high-risk patient groups, underscoring the broader implications of our findings.

## Materials and methods

### Acquisition and processing of data

To investigate the relationships between TAMs and the clinical efficacy of immunotherapy, we performed a comprehensive analysis of the TME. The scRNA-seq transcriptome profiles (GSE125449) of liver cancer patients undergoing immunotherapy were retrieved from the Gene Expression Omnibus (GEO) database. Next, data preprocessing was performed using the **Seurat (v4.3.0)** R package, which included cell and gene filtering, data normalization, principal component analysis (PCA), and t-distributed stochastic neighbor embedding (t-SNE). The criteria for quality control were similar to those described by the original data contributors. Cells with more than 10% mitochondrial gene expression were excluded, while those expressing over 500 genes and with genes present in more than 5 cells were retained. To account for potential batch effects among different single-cell samples, the Harmony algorithm was applied after PCA for batch effect correction. Harmony integration was conducted using the RunHarmony function within Seurat package to ensure that biological variation, rather than technical artifacts, guided the downstream clustering and differential analysis. To identify genes with high levels of variability for downstream analyses, scRNA-seq data were normalized. High-quality scRNA-seq data were normalized to identify highly variable genes for downstream analyses. Next, we performed PCA to detect the top meaningful principal components (PCs), and the top 20 PCs were clustered using the t-SNE algorithm.

In parallel, the bulk RNA-seq data for HCC patients (n = 424) was retrieved from the TCGA database using the TCGAbiolinks package [13]. Raw count data were converted into TPM format. Corresponding clinical information, including survival data, follow-up duration, and cancer stage, was retrieved from the TCGA. Patients without complete survival or TNM staging data were excluded, resulting in a final cohort of 424 hepatocellular carcinoma (HCC) patients for subsequent analyses to develop the predictive model. To evaluate the robustness of our predictive model, we downloaded six external validation datasets (GSE76427, GSE15654, GSE10141, GSE112790, GSE174570, and GSE228782) from GEO database. To correct for batch effects across these external bulk transcriptome datasets, we used the Combat function from the sva R package (version 3.54.0), which employs an empirical Bayes framework to adjust for non-biological variation between batches. This step ensured consistency in downstream differential expression and prognostic analyses across datasets of different origins. Comprehensive details for the datasets utilized in this analysis are presented in Table 1.

**Table 1:** List of datasets used in this study.

Datasets Name	Source	No. Of Sample	Sequencing Type	PMID
GSE125449	GEO	19	Single-cell RNAseq	39932456
GSE76427	GEO	167	Bulk RNAseq	29117471
GSE15654	GEO	216	Bulk RNAseq	23333348
GSE10141	GEO	80	Bulk RNAseq	18923165
GSE112790	GEO	198	Bulk RNAseq	30598371
GSE174570	GEO	114	Bulk RNAseq	35302601
GSE228782	GEO	83	Bulk RNAseq	38072306
TCGA-LIHC	TCGA	424	Bulk RNAseq	–

Furthermore, TAMs-associated marker gene sets were obtained from the study by Jiang et al. Additionally, 50 hallmark gene sets were retrieved from the MSigDB using the “h.all.v2023.1.Hs.symbols.gmt” file. KEGG pathway sets were acquired from the “c2.cp.kegg.v7.4.symbols.gmt” file for subsequent gene set enrichment analysis (GSEA).

### Identification of differentially expressed genes (DEGs) related to immunotherapy response and TAMs

The AUCell algorithm was used to calculate TAMs scores for individual cells in the GSE125449 dataset. To explore the association between TAMs and immunotherapy efficacy, the “FindAllMarkers” function was employed to detect DEGs between immunotherapy responders and non-responders, as well as between high- and low-TAM-score samples. A Venn diagram was then utilized to identify the intersection of DEGs associated with both immunotherapy response and TAMs, which were defined as candidate DEGs.

### Screening prognostically relevant genes in the TCGA-LIHC cohort

To further identify prognostically relevant genes among the candidate DEGs, we utilized the TCGA-LIHC dataset, which includes comprehensive survival information, as the HCC datasets from the GEO database lack such data. Univariate Cox regression analysis was initially conducted to determine DEGs significantly linked to patient prognosis. Subsequently, stepwise Cox regression analysis was then applied to refine this list and identify the optimal combination of prognostic genes capable of most effectively predicting patient survival. This approach aimed at enhancing the robustness and clinical relevance of the final prognostic gene set, providing a foundation for constructing predictive models with high accuracy and interpretability.

### Construction of a prognostic prediction model in TCGA-LIHC cohort

To evaluate the prognostic predictive capability of the optimal combination of prognostic genes in HCC patients, we constructed a logistic regression-based prognostic model using the TCGA-LIHC dataset. The predictive score formula was calculated by incorporating gene expression levels and their corresponding logistic regression coefficients. Predictive Score = (Exp_1_*Coef_1_) + (Exp_2_*Coef_2_) +**...**+ (Exp_n_*Coef_n_). Patients were divided into high-risk and low-risk groups according to their predictive scores. Overall survival (OS) differences between the groups were analyzed using Kaplan-Meier survival curves and log-rank tests with the “survival” R package. To further assess the model’s predictive performance, time-dependent ROC curves were generated, and AUC values were determined using the “timeROC” R package.

To assess the robustness and generalizability of the predictive model, we tested its performance on external validation datasets. By applying the same predictive scoring formula, patients were similarly classified into low-risk and high-risk groups. Kaplan-Meier analysis, log-rank tests, and time-dependent ROC curve analysis were repeated to confirm the model’s stability and consistency in predicting survival outcomes across independent datasets. This comprehensive validation ensured that the model retained high predictive accuracy across diverse patient populations.

### Tumor microenvironment and drug sensitivity analysis

To elucidate the potential mechanisms underlying the prognostic value of the model, we performed immune infiltration analysis using the CIBERSORT [14] and ssGSEA [15] algorithms. Specifically, CIBERSORT estimated the relative proportions of 22 distinct immune cell types within the TME of HCC patients. Concurrently, the ssGSEA algorithm was applied to evaluate the infiltration of 24 immune cell types. Both CIBERSORT and ssGSEA analyses were performed using the exp2cell function from the SMDIC R package, with 100 iterations of permutation applied to ensure robustness of the immune cell infiltration estimates. Subsequently, the relationship between the expression of prognosis-related gene and the abundance of immune cell types was assessed using person correlation analysis. Additionally, by utilizing the Genomics of Drug Sensitivity in Cancer (GDSC) database, we predicted the sensitivity of HCC to various chemotherapeutic agents based on the expression levels of genes by oncoPredict algorithm. This analysis aimed to provide insights into whether the signature genes could be used as potential targets for HCC therapy.

### Statistical Analysis

All data processing and statistical analyses were conducted using R software (version 4.0.2). For the purpose of comparing continuous variables between two groups with a normal distribution, the t-test was utilized, otherwise the Mann-Whitney U test was used. The Chi-square test or Fisher’s exact test were applied for analyzing categorical variables. Furthermore, survival analysis was conducted using the R package “survival”, accompanied by the plotting of Kaplan-Meier curves to visually depict survival between distinct groups, along with the application of the log-rank test for the purpose of evaluating statistical significance. P < 0.05 was considered as statistical significance.

## Results

**Fig 1:**
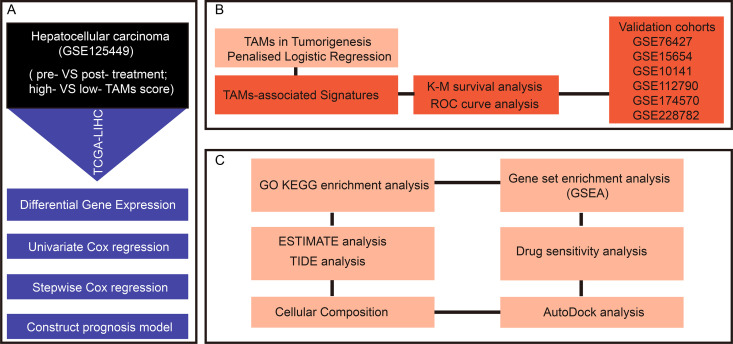
The flowchart of this study.

In the tumor microenvironment, macrophages play a complex role. They are not only capable of clearing pathogens and dead cells through phagocytosis but also regulate the immune response by secreting cytokines. A growing corpus of research findings has demonstrated a close correlation between the expression patterns of specific genes belonging to the macrophages and the efficacy of cancer immunotherapy, as well as the development and progression of HCC. However, the underlying biological mechanisms remain poorly understood. To this end, the present study adopted bioinformatics approaches for identification of TAM-associated features relevant to immunotherapy and used them to develop a prognostic prediction model for HCC. The detailed workflow is illustrated in Fig 1. Specifically, we first obtained single-cell sequencing data of HCC patients treated with immunotherapy from the GEO database. By performing differential expression analysis between pre- and post-treatment patients, along with groups with high and low TAM levels, we identified a set of DEGs linked to TAMs and immunotherapy. Based on these genes’ expression, we conducted univariate Cox regression analysis followed by stepwise Cox regression to identify the optimal gene list related with HCC prognosis. Finally, we applied logistic regression to construct a predictive model for HCC prognosis, which effectively stratifies patient outcomes. Furthermore, the present investigation sought to elucidate the functional contributions of these genes within the complex interplay of the TME, particularly regarding their potential influence on immune evasion strategies and the response to therapeutic interventions. Through these analyses, our objective is to provide novel targets and strategies for improving prognosis in HCC.

### Decoding the impact of tumor-associated macrophages on immunotherapy

**Fig 2:**
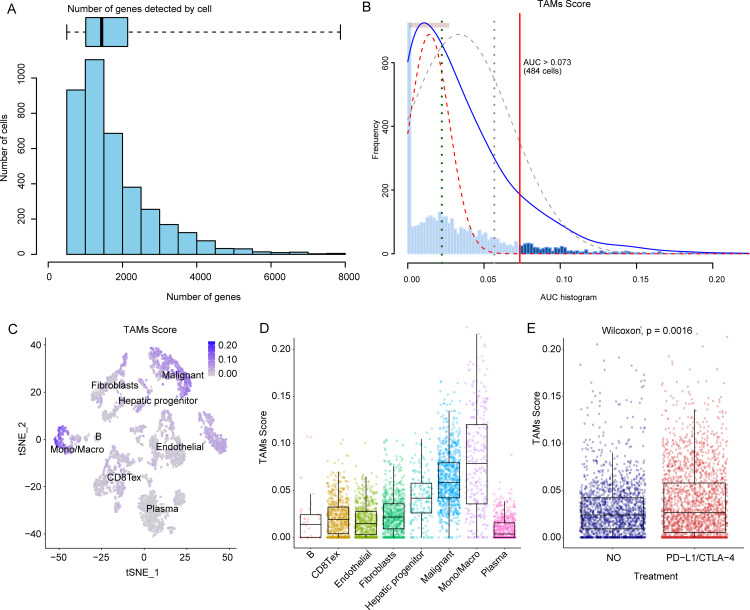
Calculating the TAMs-associated score. (A) The number of genes detected by cell; (B) The AUC histogram obtained from AUCell algorithm; (C) t-SNE analysis of cell types; (D) Distribution of TAMs score in different cell types; (E) Distribution of TAMs score between HCC patients receiving and not receiving immunotherapy.

We commenced our analysis by evaluating the association between tumor-associated macrophages and the effectiveness of immunotherapy in the GSE125449 HCC dataset. Utilizing the AUCell algorithm, we determined TAMs scores for individual cells (Fig 2A–B). The results indicated that TAMs scores were notably elevated in monocytes, macrophages, and tumor cells, with monocytes and macrophages showing significantly higher scores than other cell types (with P values < 0.05, Fig 2C–D). This suggests a potentially pivotal role for TAMs in hepatocellular carcinoma. Subsequently, we examined the variation in TAM scores between pre- and post-immunotherapy samples. Strikingly, there was a marked increase in TAM scores among patients who received immunotherapy (P value = 0.0016, Fig 2E). This observation points to a likely connection between TAMs and the immunotherapy response, suggesting that TAMs may contribute to the dynamics of the immunotherapy process.

Previous study has demonstrated that the polarization states of TAMs, specifically the M1 and M2 phenotypes, play a pivotal role in tumor progression and therapeutic response [16–18]. Classically activated M1 macrophages are generally characterized by their pro-inflammatory and tumoricidal properties, including the secretion of cytokines such as IL-12 and TNF-α, which promote antigen presentation and enhance cytotoxic T cell activation. In contrast, alternatively activated M2 macrophages—commonly regarded as the predominant phenotype of TAMs in many solid tumors—are associated with tumor-promoting functions. Given the functional heterogeneity of TAMs, we sought to further investigate their polarization dynamics in the context of cancer immunotherapy. Specifically, we employed the AddModuleScore function from the Seurat package to quantify M1 and M2 polarization states based on previously validated marker gene sets. Our analysis revealed a transcriptional bias toward M2-like macrophage polarization in patients who had not undergone immunotherapy, indicative of a more immunosuppressive TME. In contrast, patients treated with immune checkpoint blockade (ICB) exhibited a more balanced or even M1-skewed TAM profile, suggesting that immunotherapy may reshape macrophage polarization toward a more pro-inflammatory and immunostimulatory state (S1 Fig). These findings underscore the potential of TAM polarization status not only as a biomarker for immunotherapeutic responsiveness but also as a targetable axis for enhancing treatment efficacy.

**Fig 3:**
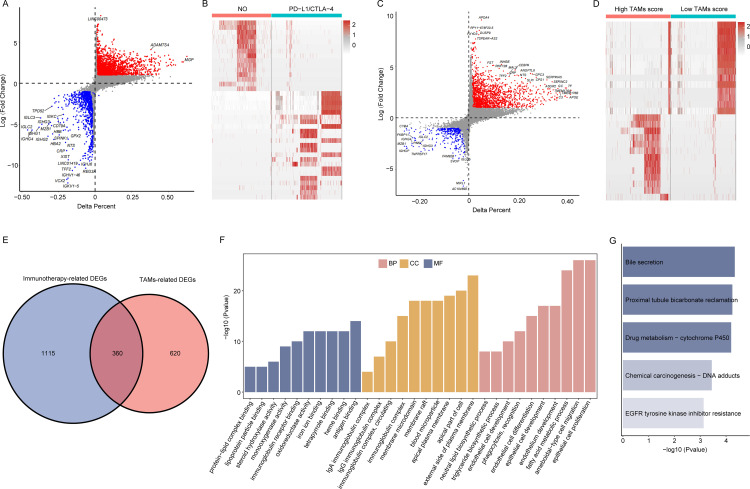
Identifying immunotherapy and TAMs related genes. (A) The volcano plot of gene expressed differences between patients receiving and not receiving immunotherapy; (B) Heatmap of DEGs for patients receiving and not receiving immunotherapy; (C) The volcano plot of gene expressed differences between high- and low- TAMs score patients; (D) Heatmap of DEGs for patients with high- and low- TAMs score; (E) Immunotherapy and TAMs related DEGs Venn diagram; (F) GO functional enrichment analysis of DEGs; (G) KEG functional enrichment analysis of DEGs.

To delve into the underlying biological mechanisms, we initially conducted a differential gene expression analysis of samples before and after immunotherapy, pinpointing genes that exhibited significant changes in expression levels. Through this analysis, we identified a total of 1115 immunotherapy-associated differentially expressed genes (Fig 3A–B). Our previous findings suggested that tumor-associated macrophages (TAMs) may be involved in the response to immunotherapy. Patients were subsequently categorized into two groups according to median TAM scores, herein designated as low and high TAM score groups, with the objective of identifying DEGs between these groups. This analysis uncovered 620 DEGs related to macrophages (Fig 3C–D). To further pinpoint genes that are both related to immunotherapy and macrophages, we intersected the set of immunotherapy-related DEGs with the TAMs-related DEGs, resulting in the identification of 360 overlapping genes (Fig 3E), which included genes from the immunoglobulin family (such as IGHG1, IGHG2, IGHG3, IGHG4) and the cytochrome P450 family (like CYP2A7, CYP4A11, CYP2A6). Immunoglobulins represent a class of proteins that fulfill a pivotal function within the immune system, primarily responsible for recognizing and binding to antigens, thereby triggering an immune response. The cytochrome P450 family, on the other hand, consists of a large group of enzymes containing heme groups, which play a significant role in drug metabolism. CYP2A7, in particular, is an important enzyme involved in the metabolic process of a variety of drugs and xenobiotics, including the biotransformation and detoxification of certain pharmaceuticals. With high expression levels in the liver, CYP2A7 significantly impacts the metabolism of many clinical drugs, thus holding substantial relevance in pharmacogenetics and personalized medicine. To further investigate the functions of these DEGs, we employed Gene Ontology (GO) and KEGG enrichment analysis. The GO analysis identified significant enrichment in three aspects: biological processes, cellular components, and molecular functions, involving key biological processes such as the immunoglobulin complex, antigen binding, and amoeboid-type cell migration (Fig 3F). Pathway enrichment analysis indicated that the DEGs were predominantly related to drug metabolism and chemical carcinogenesis (Fig 3G). These analyses yielded a more profound comprehension of the potential mechanisms through which TAMs mediate their effects in immunotherapy, providing a theoretical foundation for subsequent clinical research.

### Constructing a prognostic model for hepatocellular carcinoma

**Fig 4:**
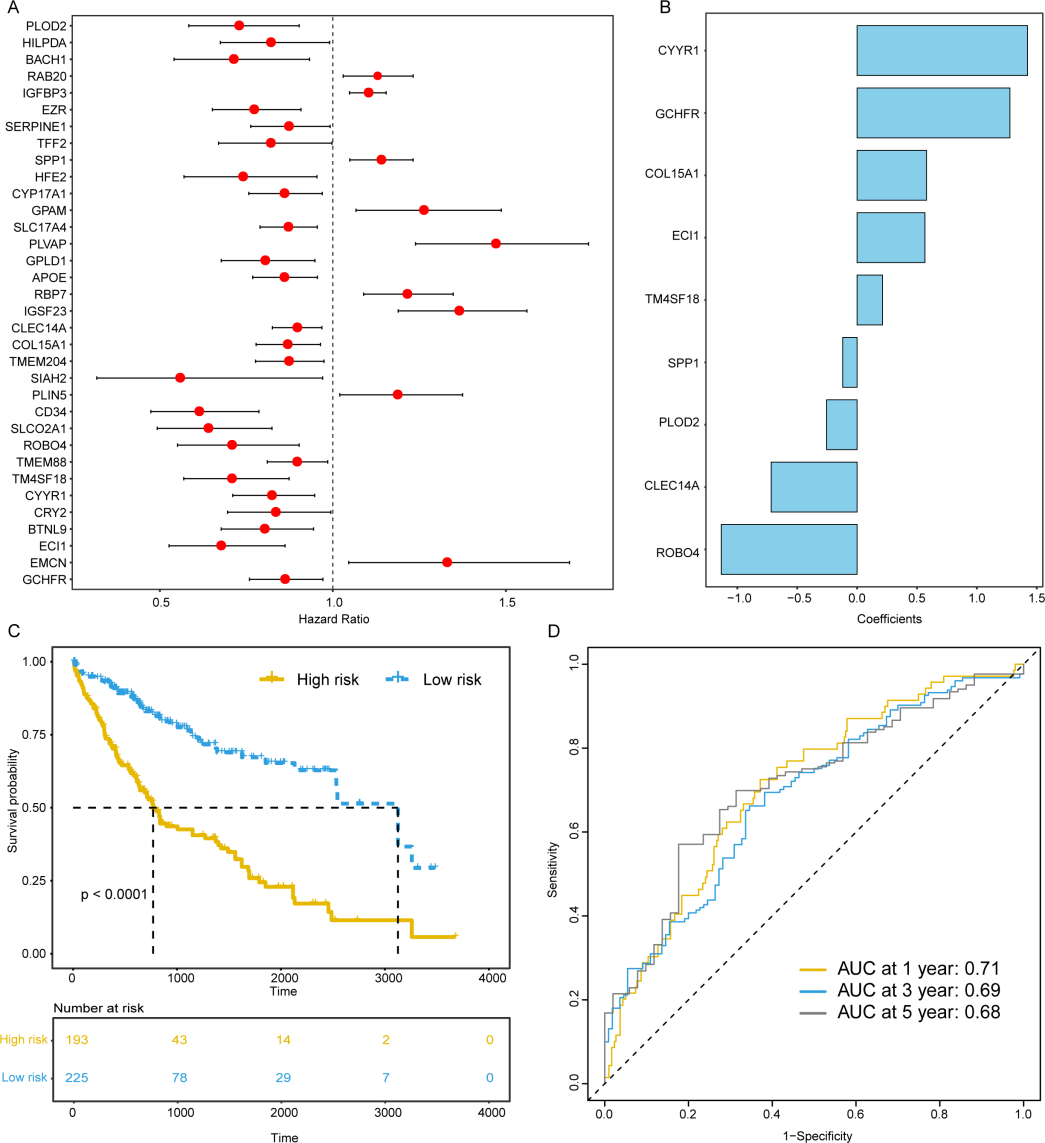
Constructing the prognostic model for HCC. (A) Forest plot of prognosis-related genes; (B) The histogram of logistic regression coefficients for signature genes; (C) Kaplan-Meier survival curve between high-risk and low-risk sample groups; (D) Time-dependent ROC curve of the model for predicting patients’ prognosis.

Subsequently, we further identified key genes among these differentially expressed genes that are linked to HCC prognosis. In light of the paucity of clinical information in the GSE125449 dataset, we proceeded with subsequent HCC prognosis-related analyses using the TCGA-LIHC dataset. Initially, we employed univariate Cox regression analysis to pinpoint genes within the DEGs that correlate with patient prognosis, resulting in the identification of 34 genes associated with prognosis, including 9 prognostic risk factors and 25 protective factors (Fig 4A). To discern the optimal set of genes for predicting patient outcomes, stepwise regression analysis was conducted, leading to the identification of 9 genes, such as CLEC14A, TM4SF18, PLOD2, ROBO4, CYYR1, GCHFR, ECI1, SPP1, and COL15A1 (Fig 4B and Table 2). CLEC14A has been characterized as a tumor endothelial marker that plays a pivotal role in pathological angiogenesis [19]. Mechanistically, it modulates signaling pathways mediated by vascular endothelial growth factor receptors VEGFR-2 and VEGFR-3, thereby contributing to endothelial sprouting and maintenance of vascular integrity [20]. Notably, the interaction between CLEC14A and multimerin-2 (MMRN2) has been shown to facilitate angiogenic processes, and pharmacological disruption of this interaction significantly suppresses sprouting angiogenesis and impedes tumor growth, underscoring its potential as a therapeutic target in anti-angiogenic strategies. CYYR1 (Cysteine and Tyrosine-Rich 1), although less well characterized, has been implicated in the pathogenesis of several malignancies, including breast, ovarian, and colorectal cancers [21–23]. The Aberrant expression of CYYR1 has been associated with tumor progression, suggesting that it may function as an oncogenic factor contributing to tumorigenesis and disease advancement.

**Table 2:** Coefficients of the genes included in the prognostic model.

Gene Symbol	Description	Coefficient (95%CI)
CYYR1	Cysteine and tyrosine-rich protein 1	1.422 (2.395, 0.486)
GCHFR	GTP cyclohydrolase I feedback regulatory protein	1.276 (2.069, 0.522)
COL15A1	Collagen type XV alpha 1 chain	0.580 (0.922, 0.248)
ECI1	Enoyl-CoA isomerase 1	0.567 (0.954, 0.1926)
TM4SF18	Transmembrane 4 L6 family member 18	0.212 (0.774, −0.328)
SPP1	Secreted phosphoprotein 1 (osteopontin)	−0.120 (−0.043, −0.199)
PLOD2	Procollagen-lysine,2-oxoglutarate 5-dioxygenase 2	−0.255 (−0.005, −0.512)
CLEC14A	C-type lectin domain family 14 member A	−0.717 (−0.168, −1.283)
ROBO4	Roundabout guidance receptor 4	−1.134 (−0.374, −1.923)

We then utilized logistic regression analysis to construct an HCC prognosis model using the expression data of the above 9 genes, calculating a Predictive Score for each sample: Predictive Score = CYYR1 * 1.422 + GCHFR * 1.276 + COL15A1 * 0.580 + ECI1 * 0.567 + TM4SF18 * 0.212 - SPP1 * 0.120 - PLOD2 * 0.255 - CLEC14A * 0.717 - ROBO4 * 1.134 (Table 2). Employing ROC curve analysis, we determined an optimal threshold value, which was used to stratify samples into two groups. Subsequently, a comparison was performed to ascertain the disparities in overall survival between these two distinct groups. The utilization of Kaplan-Meier survival analysis yielded a discernible outcome: low-risk samples exhibited a marked increase in overall survival periods when contrasted with high-risk samples (median OS, 1.814 months versus 1.425 months, P < 0.0001, Fig 4C). Subsequently, time-dependent ROC curve analysis indicated that the model achieved an AUC of 0.71 for predicting patient survival within one year (Fig 4D).

### Comparison of the 9-gene prognostic model with existing biomarkers

To benchmark the predictive utility of our 9-gene prognostic model, we compared its performance with several previously reported HCC prognostic biomarkers. These included the expression levels of SMPD3 [24], GPC3 [5], CD274, CTLA4, LAG3, and HAVCR2 [4], as well as a 12-gene prognostic signature developed by Yan et al. [25]. We evaluated each model’s ability to predict 1-year overall survival using the area under the ROC curve (AUC) and assessed their stratification power using Kaplan-Meier survival analysis. As shown in S2A Fig, our 9-gene model achieved the highest AUC (0.71), outperforming SMPD3 (0.52), GPC3 (0.53), CD274 (0.48), CTLA4 (0.52), LAG3 (0.52), HAVCR2 (0.56), and the 12-gene signature (0.56). Kaplan-Meier analysis further demonstrated that only the 12-gene signature, among the biomarkers, was able to separate patients into risk subgroups with statistically significant survival differences. However, the stratification achieved by our 9-gene model was more robust, with greater separation of survival curves and a more significant log-rank P value (S2B–H Fig). These findings collectively indicate that our model provides superior prognostic discrimination and may offer greater clinical utility than existing single-gene and multi-gene biomarkers in HCC.

### Assessing the differences in immune infiltration between high- and low-risk samples

**Fig 5:**
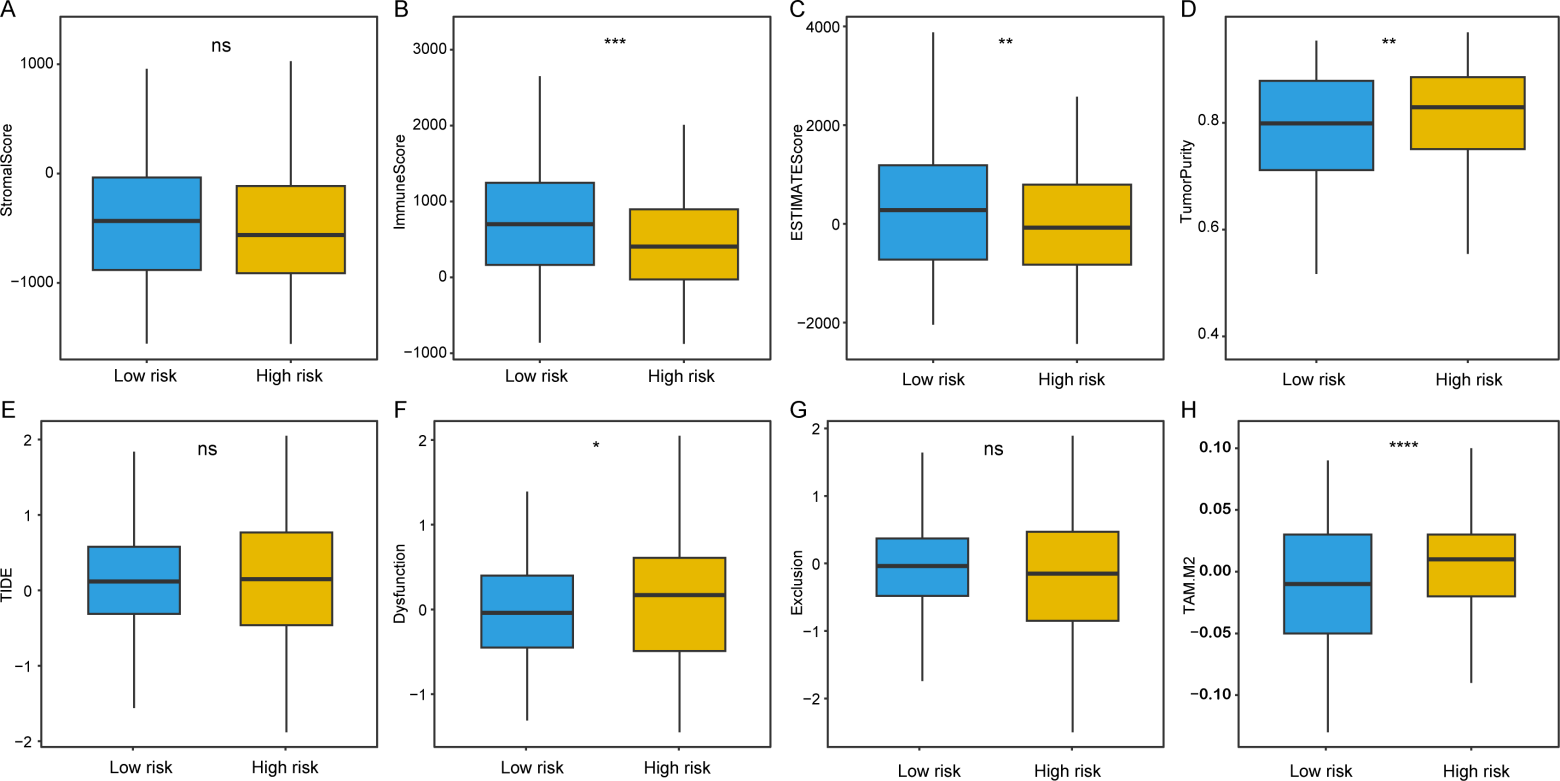
Comparison of immune features between high-risk and low-risk sample groups. Comparison of stromal score (A), immune score (B), ESTIMATE score (C), tumor purity (D), TIDE (E), Dysfunction (F), Exclusion (G), TAM.M2 score between high-risk and low-risk sample groups.

After this, we proceeded to assess the disparities in immune infiltration between the high and low-risk groups. Initially, we calculated several scores—stromal, immune, ESTIMATE, and tumor purity—applying the ESTIMATE algorithm. Our results revealed that low-risk samples exhibited higher stromal, immune, and ESTIMATE scores, along with lower tumor purity (Fig 5A–D). We then conducted a TIDE analysis on the dataset to further investigate the presence of Tumor Immune Dysfunction and Exclusion, indicating that high-risk samples were more prone to exhibit tumor immune dysfunction and exclusion (Fig 5E–G). Additionally, a significant decrease in the scores related to tumor-associated M2 macrophages was observed in low-risk samples (P < 0.05, Fig 5H). M2 macrophages, a subtype of tumor-associated macrophages, have been shown to exert a multifaceted function in cancer progression and therapy response. They may contribute to the malignancy of tumors by promoting angiogenesis, tumor cell growth, and metastasis via releasing a range of cytokines and signaling molecules. In some contexts, M2 macrophages may suppress anti-tumor immune responses by suppressing the activity of T cells and natural killer (NK) cells, potentially leading to a poorer prognosis for HCC patients.

**Fig 6:**
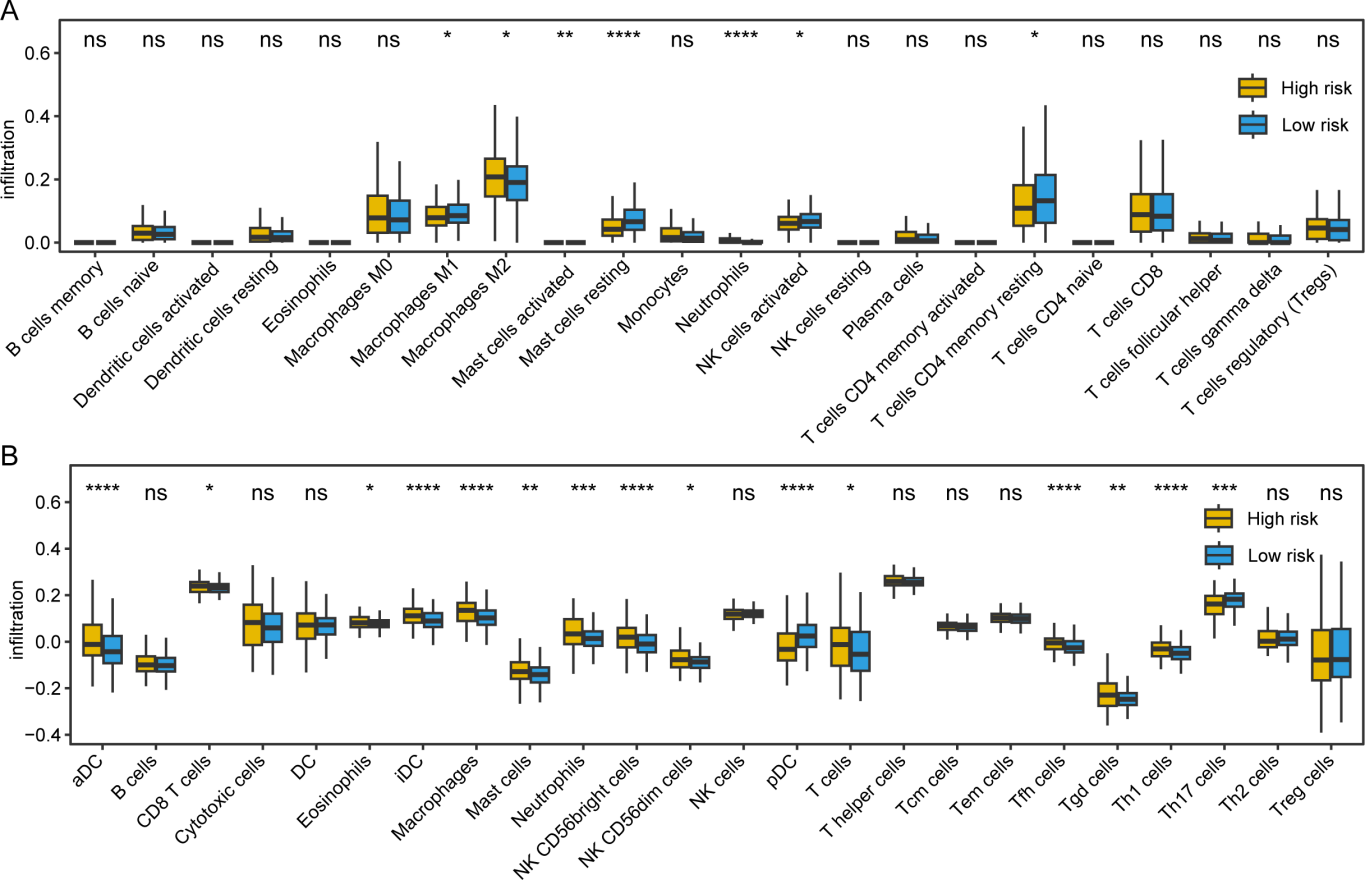
Comparison of immune cell infiltration between high-risk and low-risk sample groups. (A) Comparison of immune cell infiltration between high-risk and low-risk sample groups estimated by CIBERSORT algorithm; (B) Comparison of immune cell infiltration between high-risk and low-risk sample groups estimated by ssGSEA algorithm.

To enhance our comprehension of the mechanisms driving these differences, we took a meticulous examination of TME between patients with distinct risk scores. The results indicated that in low-risk samples, there was a higher infiltration level of regulatory T cells (Tregs) and dendritic cells (DCs), which may enhance the anti-tumor immune response. In contrast, high-risk samples exhibited lower infiltration levels of tumor-infiltrating lymphocytes (TILs), potentially associated with poorer prognosis. Additionally, we observed higher expression levels of pro-inflammatory cytokines, including IL-6 and TNF-α in high-risk samples, which may reflect an inflammatory state within the tumor microenvironment. These findings suggest that differences in immune infiltration may be closely related to the composition and functionality of immune cells within the TME, thereby influencing HCC clinical outcomes. We employed the CIBERSORT and ssGSEA algorithms to assess relative immune cell abundance. By comparing the immune infiltration among individuals with low-risk and high-risk score subgroups, we identified significant differences in certain immune cell types. For CIBERSORT, consistent with prior findings, elevated levels of M2 macrophages were observed in high-risk groups, while increased infiltration of M1 macrophages was noted in low-risk samples (Fig 6A). For ssGSEA, we discovered that high-risk patients enriched in macrophages, cytotoxic cells, mast cells, and neutrophils (Fig 6B). Moreover, we found that the immune checkpoint-related genes were under-expression in high-risk samples. Subsequently, an in-depth investigation was undertaken to elucidate the relationship between signature genes expression and sample immune composition. The result of person correlation analysis demonstrated a significant correlation between signature genes expression and patient immune infiltration. These discoveries suggest that differences in immune infiltration may be closely associated with the composition and status of immune cells in TME, thus affecting the clinical outcomes of HCC patients.

**Fig 7:**
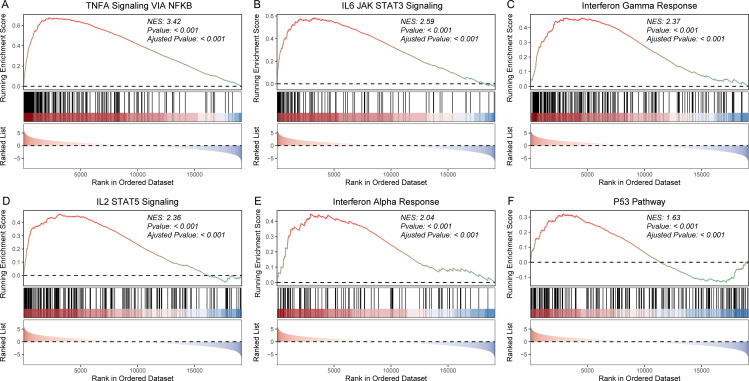
GSEA analysis of HALLMARK gene sets between high-risk and low-risk sample groups. GSEA plots of TNFA signaling via NFKB (A); IL6 JAK STAT3 signaling (B); interferon gamma response (C); IL6 IL2 STAT5 signaling (D); interferon alpha response (E); and P53 pathway (F).

**Fig 8:**
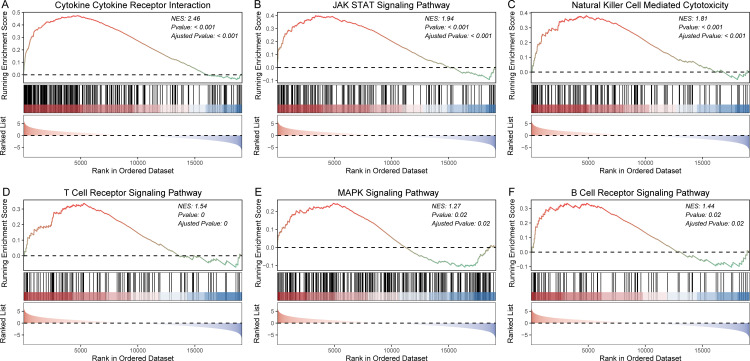
GSEA analysis of KEGG pathways between high-risk and low-risk sample groups. GSEA plots of cytokine cytokine receptor interaction (A); JAK STAT signaling pathway (B); natural killer cell mediated cytotoxicity (C); T cell receptor signaling pathway (D); MAPK signaling pathway (E); and B cell receptor signaling pathway (F).

To further validate these results, we also performed GSEA to explore biological pathways associated with HCC prognosis. Our analysis revealed significant differences in biological processes associated with cancer development between distinct patient subgroups. Specifically, low-risk samples exhibited significant involvement in hallmark pathways such as TNFA signaling, IL6-JAK-STAT3, P53 pathway, and so on (Fig 7 and S1 Table). To further validate our findings, GSEA analysis was also performed based on the KEGG pathway gene sets. The results showed that low-risk samples exhibited significant involvement in cytokine-cytokine receptor interaction, JAK STAT signaling pathway, T/B cell receptor signaling pathway, and MAPK signaling pathway (Fig 8 and S2 Table). In summary, these findings indicate that low-risk patients exhibit activation of immune-related and tumor-suppressive pathways, correlating with favorable prognosis, while high-risk patients show suppressed immune activity and enhanced oncogenic signaling, contributing to poor outcomes. These insights underscore the molecular basis of HCC prognosis and highlight potential therapeutic targets.

### Drug sensitivity analysis

**Fig 9:**
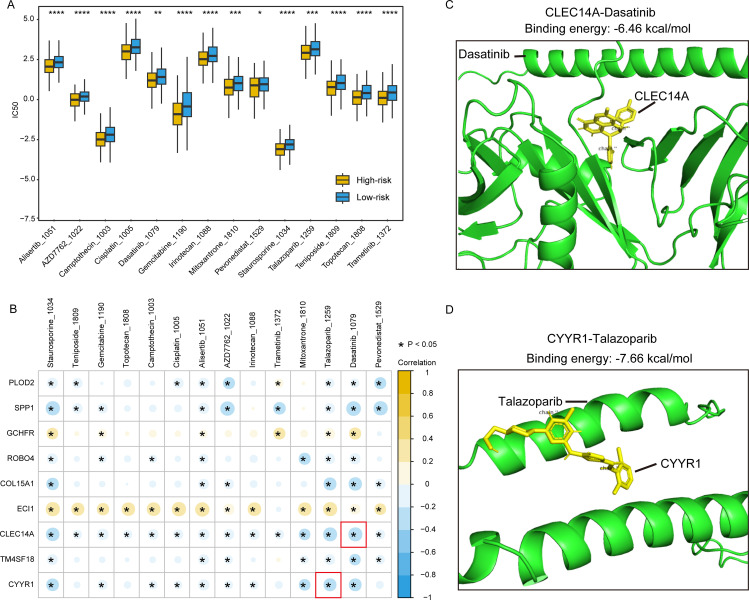
Drug sensitivity analysis. (A) Distribution of drug sensitivity between high-risk and low-risk HCC patients; (B) Correlation heatmap between the expression of signature genes and drug sensitivity; **(C)** 3-D schematic view of AutoDock between gene CLEC14A and drug dasatinib; **(D)** 3-D schematic view of AutoDock between gene CYYR1 and drug talazoparib.

Further investigation into the relationships between the risk model and drug sensitivity was undertaken by employing the oncoPredict algorithm to calculate IC50 values for drugs corresponding to TCGA-LIHC dataset samples against the GDSC database. oncoPredict predicts patient responses to various drugs based on specific gene expression patterns. Subsequently, we identified disparities in drug sensitivity among high-risk and low-risk patients (Fig 9A), revealing that 14 drugs exhibited significant differences in IC50 values between these groups, such as gemcitabine, trametinib, and dasatinib (P < 0.05, Fig 9A). Dasatinib, an oral multikinase inhibitor used to treat Philadelphia chromosome (Ph) -positive leukemia, has also been found to be useful for HCC [26]. We then performed a correlation analysis between the signature genes expression and drug susceptibility, uncovering that the expression of genes CLEC14A and CYYR1 was significantly negatively correlated with sensitivity to all drugs (Fig 9B), suggesting their potential key roles in HCC progression and treatment response. Specifically, we also found that the expression of the gene CLEC14A exhibited the strongest correlation with sensitivity to the drug dasatinib, while the expression of CYYR1 showed the highest correlation with sensitivity to talazoparib. To further investigate the possible application for signature genes as therapeutic targets for these drugs, molecular docking examination was conducted. Utilizing the AutoDock software, our findings indicated that the optimal binding conformation of CLEC14A with dasatinib demonstrated a high binding affinity, with a binding energy of −6.46 kcal/mol (Fig 9C). Similarly, the optimal binding conformation of CYYR1 with talazoparib manifested an impressive binding affinity, exhibiting a binding energy of −7.66 kcal/mol (Fig 9D). These findings suggested that signature genes we identified could serve as potential therapy targets and offer clinical guidance in the development of personalized treatment strategies.

### Validation of the model’s predictive performance

**Fig 10:**
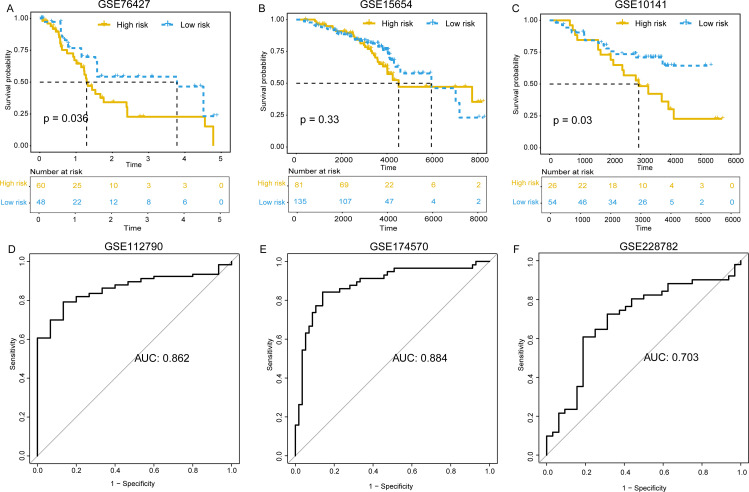
Validating the predictive and prognostic performances of model across cohorts. Kaplan-Meier survival curve between high-risk and low-risk sample groups in GSE76427 cohort (A), GSE15654 cohort (B), GSE10141 cohort (C); ROC curve of model for predicting HCC in GSE112790 cohort (D), GSE174570 cohort (E), GSE228782 cohort (F).

To validate the predictive capacity for our model, we first applied it to external validation datasets for HCC. We began by assessing the model’s prognostic capabilities in HCC datasets with clinical information, including GSE76427, GSE15654, and GSE10141. Predictive scores were calculated and an optimal threshold determined for all samples according to the ROC curve. The samples were then categorized as low-risk and high-risk groups. Subsequently, we conducted survival analysis to ascertain the disparities in prognosis among low-risk and high-risk subgroups. Our results indicated that patients with high-risk scores exhibited notably poorer clinical outcomes against those with low-risk scores across all three cohorts, as clearly depicted by the Kaplan-Meier survival curves (P < 0.05, Fig 10A–C), confirming the model’s accuracy and reliability in predicting HCC patient outcomes. Furthermore, we also downloaded three additional HCC datasets without prognostic information from the GEO database. We then applied our predictive model to these external validation datasets to further verify the model’s predictive power for HCC. The ROC curve analysis demonstrated that the model maintained a high level of performance in predicting HCC across the GSE112790, GSE174570, and GSE228782 datasets, with AUC values of 0.862 for GSE112790, 0.884 for GSE174570, and 0.703 for GSE228782 (Fig 10D–F).

## Discussion

In this study, we presented a comprehensive integrative analysis by combining scRNAseq data with bulk transcriptomic profiles from TCGA-LIHC to elucidate the prognostic significance of tumor-associated macrophages in HCC prognosis. By employing univariate Cox regression analysis and stepwise Cox regression analyses, we identified nine TAM-related prognostic genes and constructed a predictive model based on their expression profiles. This model effectively stratified HCC patients into high- and low-risk subgroups with distinct survival outcomes across multiple cohorts, as demonstrated by Kaplan–Meier curves and time-dependent ROC analyses, underscoring its clinical potential.

Beyond its prognostic utility, our model offers mechanistic insights into how TAMs orchestrate the immunological landscape of HCC. TAMs represent a phenotypically plastic population capable of polarizing into pro-inflammatory M1-like or immunosuppressive M2-like states in response to microenvironmental cues. In HCC, a predominant M2-like polarization contributes to an immunosuppressive milieu through multiple mechanisms, including the secretion of anti-inflammatory cytokines (e.g., IL-10, TGF-β), promotion of regulatory T cell recruitment, suppression of cytotoxic T lymphocyte function, and enhancement of angiogenesis and extracellular matrix remodeling. These mechanisms collectively enable tumor progression and immune evasion. In our study, we observed that high-risk patients displayed increased expression of M2-associated markers and reduced infiltration of tumor-infiltrating lymphocytes (TILs), supporting the hypothesis that both the quantity and polarization state of TAMs critically shape immune responsiveness and clinical outcomes. Notably, patients receiving immune checkpoint blockade exhibited a more balanced or M1-skewed macrophage profile, suggesting that TAM reprogramming may enhance immunotherapeutic efficacy in HCC [27,28].

Moreover, drug sensitivity analysis and in silico docking suggest several of the identified genes (e.g., CLEC14A, CYYR1) may serve as potential therapeutic targets. CLEC14A has been implicated in tumor angiogenesis and endothelial cell adhesion [19,20,29], while CYYR1 is reportedly dysregulated in several malignancies including breast and ovarian cancers [21–23]. However, we acknowledge that the lack of experimental validation of these targets is a limitation of our study. Given the retrospective and computational nature of this work, and current constraints in experimental capacity, we have not conducted functional assays. Nevertheless, we believe our findings provide a strong foundation for future in vitro or in vivo validation studies, which will be critical for confirming the translational relevance of our candidate genes.

Moreover, the model’s compact gene set offers practical advantages for clinical translation, potentially enabling its application via qPCR panels, RNA-seq diagnostics, or immunohistochemistry to support risk stratification and therapeutic decision-making. This enhances the feasibility of integrating the model into clinical workflows for HCC management.

In conclusion, our study highlights the prognostic value of TAM-related transcriptional programs in HCC and their potential interplay with immune responses and therapy. The proposed model offers a feasible tool for patient stratification and hypothesis generation in translational oncology. Future work should focus on prospective validation and mechanistic dissection of key candidate genes and pathways in experimental systems.

## Supporting information

S1 Fig Distribution of M1 scores between immunotherapy-treated and immunotherapy-naïve patients. **(A)** Comparison of M1 polarization scores between immunotherapy-treated and untreated samples. **(B)** Comparison. of M2 polarization scores between immunotherapy-treated and untreated samples. **(C)** t-SNE plot depicting the distribution of M1 polarization scores in immunotherapy-treated versus untreated samples. **(D)** t-SNE plot depicting the distribution of M2 polarization scores in immunotherapy-treated versus untreated samples. **(E)** Comparison of M1 polarization scores among different cell types. **(F)** Comparison of M2 polarization scores among different cell types.
(TIF)

S2 Fig Comparative prognostic performance of our 9-gene model versus established biomarkers in hepatocellular carcinoma. **(A)** ROC curves of prognostic biomarkers such as SMPD3, GPC3, CD274, CTLA4, LAG3, HAVCR2, and 12-gene signature for one year survival. **(B)** Kaplan-Meier survival analysis of OS comparing the SMPD3 high- and low-expression groups. **(C)** Kaplan-Meier survival analysis of OS comparing the GPC3 high- and low-expression groups. **(D)** Kaplan-Meier survival analysis of OS comparing the CD274 high- and low-expression groups. **(E)** Kaplan-Meier survival analysis of OS comparing the CTLA4 high- and low-expression groups. **(F)** Kaplan-Meier survival analysis of OS comparing the LAG3 high- and low-expression groups. **(G)** Kaplan-Meier survival analysis of OS comparing the HAVCR2 high- and low-expression groups. **(H)** Kaplan-Meier survival analysis of OS comparing the high- and low-risk groups classified by 12-gene signature.
(TIF)

S1 Table GSEA results based on HALLMARK gene sets. (DOCX)

S2 Table GSEA results based on KEGG pathways. (DOCX)
