# Supplementary figures and images for: Tumor-associated macrophage-based predictive and prognostic model for hepatocellular carcinoma
Source: PLoS One. 2025 Jul 2;20(7):e0325120. doi: 10.1371/journal.pone.0325120 (PMC12221018; doi:10.1371/journal.pone.0325120)

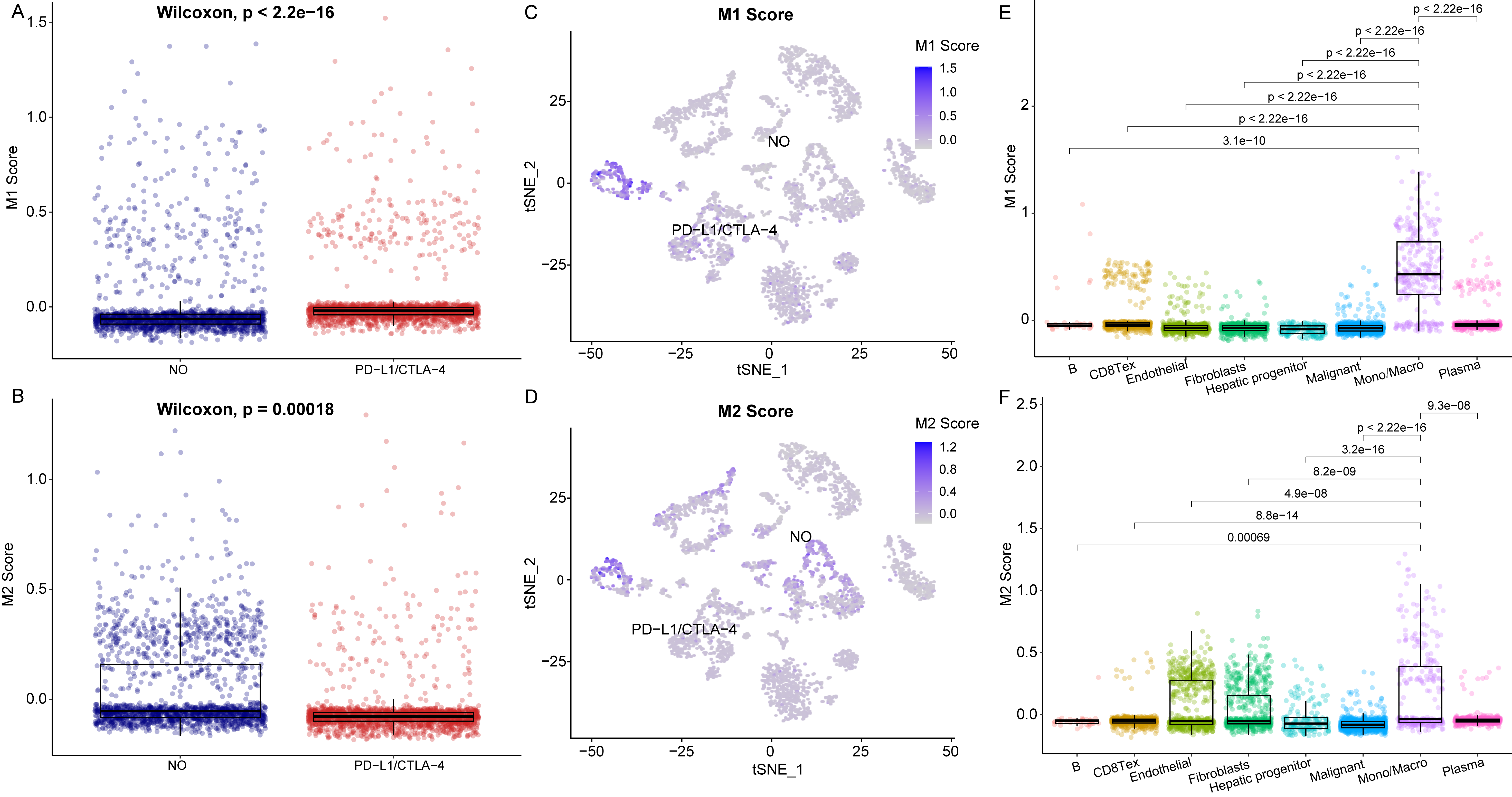

Supplement: S1 Fig — (A) Comparison of M1 polarization scores between immunotherapy-treated and untreated samples. (B) Comparison. of M2 polarization scores between immunotherapy-treated and untreated samples. (C) t-SNE plot depicting the distribution of M1 polarization scores in immunotherapy-treated versus untreated samples. (D) t-SNE plot depicting the distribution of M2 polarization scores in immunotherapy-treated versus untreated samples. (E) Comparison of M1 polarization scores among different cell types. (F) Comparison of M2 polarization scores among different cell types. (TIF) [file pone.0325120.s001.tif]

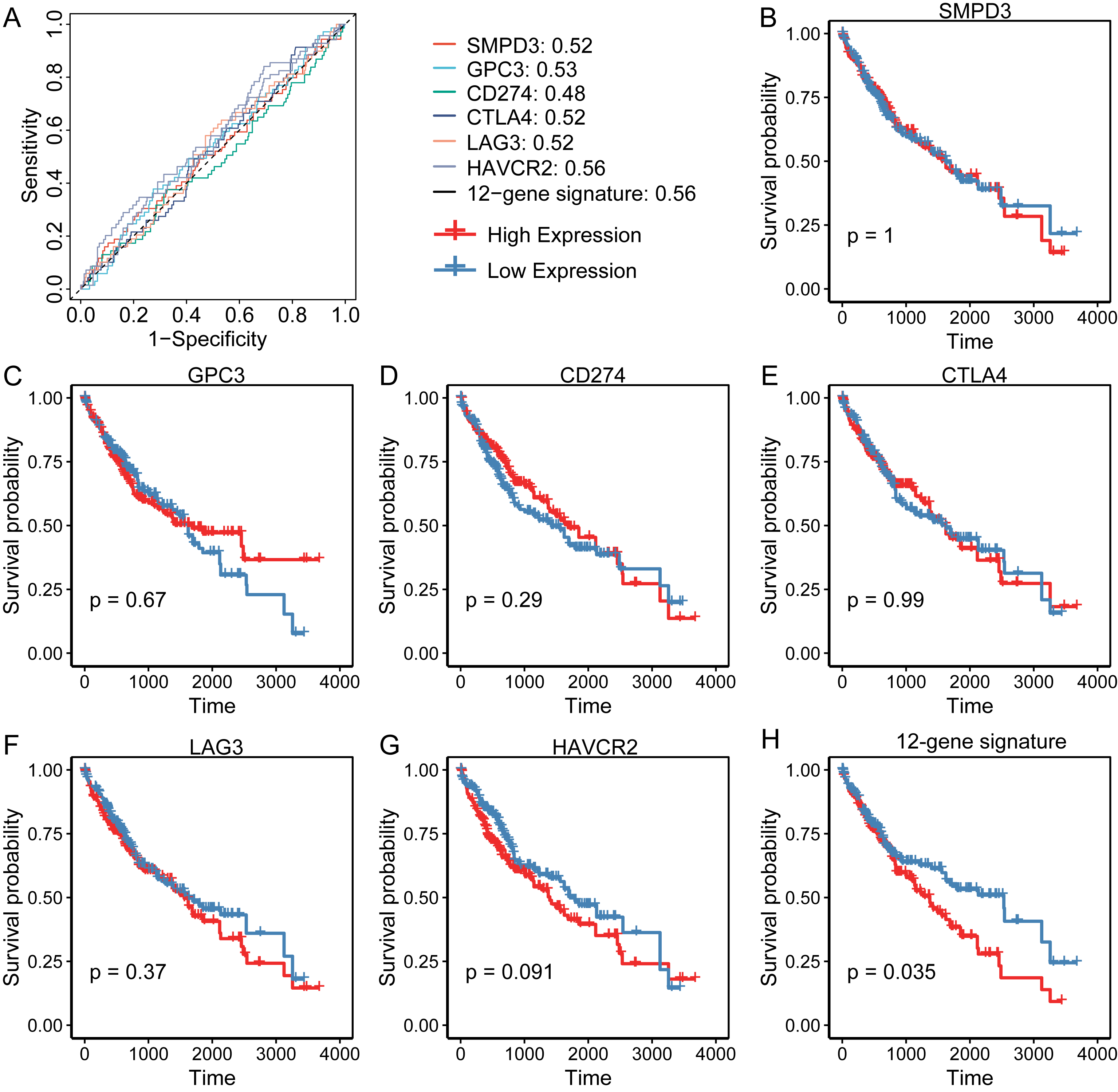

Supplement: S2 Fig — (A) ROC curves of prognostic biomarkers such as SMPD3, GPC3, CD274, CTLA4, LAG3, HAVCR2, and 12-gene signature for one year survival. (B) Kaplan-Meier survival analysis of OS comparing the SMPD3 high- and low-expression groups. (C) Kaplan-Meier survival analysis of OS comparing the GPC3 high- and low-expression groups. (D) Kaplan-Meier survival analysis of OS comparing the CD274 high- and low-expression groups. (E) Kaplan-Meier survival analysis of OS comparing the CTLA4 high- and low-expression groups. (F) Kaplan-Meier survival analysis of OS comparing the LAG3 high- and low-expression groups. (G) Kaplan-Meier survival analysis of OS comparing the HAVCR2 high- and low-expression groups. (H) Kaplan-Meier survival analysis of OS comparing the high- and low-risk groups classified by 12-gene signature. (TIF) [file pone.0325120.s002.tif]
