# Supplementary material for: Tumor-associated macrophage-based predictive and prognostic model for hepatocellular carcinoma
Source: PLoS One. 2025 Jul 2;20(7):e0325120. doi: 10.1371/journal.pone.0325120 (PMC12221018; doi:10.1371/journal.pone.0325120)
Supplement: S1 Table — (DOCX) [file pone.0325120.s003.docx]

**S1 Table.** GSEA results based on HALLMARK gene sets

| **HALLMARK** | **NES** | **P Value** |
| --- | --- | --- |
| TNFA_SIGNALING_VIA_NFKB | -3.422 | 1.000e-10 |
| INFLAMMATORY_RESPONSE | -3.176 | 1.000e-10 |
| ALLOGRAFT_REJECTION | -3.062 | 1.000e-10 |
| OXIDATIVE_PHOSPHORYLATION | 2.871 | 1.000e-10 |
| IL6_JAK_STAT3_SIGNALING | -2.598 | 1.000e-10 |
| BILE_ACID_METABOLISM | 2.588 | 1.000e-10 |
| INTERFERON_GAMMA_RESPONSE | -2.367 | 1.000e-10 |
| HYPOXIA | -2.356 | 1.000e-10 |
| IL2_STAT5_SIGNALING | -2.350 | 1.000e-10 |
| COMPLEMENT | -2.258 | 1.000e-10 |
| ADIPOGENESIS | 2.091 | 1.028e-08 |
| FATTY_ACID_METABOLISM | 2.099 | 1.673e-08 |
| KRAS_SIGNALING_UP | -2.020 | 3.142e-08 |
| PEROXISOME | 2.163 | 1.347e-07 |
| APOPTOSIS | -1.997 | 3.228e-07 |
| EPITHELIAL_MESENCHYMAL_TRANSITION | -1.860 | 2.362e-06 |
| ESTROGEN_RESPONSE_LATE | -1.847 | 3.938e-06 |
| INTERFERON_ALPHA_RESPONSE | -2.017 | 5.039e-06 |
| MTORC1_SIGNALING | -1.696 | 8.571e-05 |
| UV_RESPONSE_UP | -1.659 | 2.424e-04 |
| P53_PATHWAY | -1.627 | 3.619e-04 |
| XENOBIOTIC_METABOLISM | 1.646 | 3.913e-04 |
| ESTROGEN_RESPONSE_EARLY | -1.571 | 1.047e-03 |
| GLYCOLYSIS | -1.551 | 1.084e-03 |
| G2M_CHECKPOINT | -1.533 | 1.305e-03 |
| TGF_BETA_SIGNALING | -1.739 | 3.256e-03 |
| MYC_TARGETS_V1 | -1.423 | 6.805e-03 |
| COAGULATION | 1.404 | 1.341e-02 |
| SPERMATOGENESIS | -1.395 | 1.380e-02 |
| DNA_REPAIR | 1.414 | 1.588e-02 |
| MYC_TARGETS_V2 | -1.462 | 2.731e-02 |
| CHOLESTEROL_HOMEOSTASIS | 1.403 | 3.845e-02 |
