# Supplementary material for: Tumor-associated macrophage-based predictive and prognostic model for hepatocellular carcinoma
Source: PLoS One. 2025 Jul 2;20(7):e0325120. doi: 10.1371/journal.pone.0325120 (PMC12221018; doi:10.1371/journal.pone.0325120)
Supplement: S2 Table — (DOCX) [file pone.0325120.s004.docx]

**S2 Table.** GSEA results based on KEGG pathways

| **KEGG Pathway** | **NES** | **P Value** |
| --- | --- | --- |
| OXIDATIVE_PHOSPHORYLATION | 2.786 | 1.000E-10 |
| PARKINSONS_DISEASE | 2.647 | 1.000E-10 |
| PEROXISOME | 2.620 | 1.000E-10 |
| ALZHEIMERS_DISEASE | 2.482 | 1.000E-10 |
| CYTOKINE_CYTOKINE_RECEPTOR_INTERACTION | -2.480 | 1.000E-10 |
| HUNTINGTONS_DISEASE | 2.237 | 1.295E-10 |
| LEISHMANIA_INFECTION | -2.600 | 1.590E-10 |
| GRAFT_VERSUS_HOST_DISEASE | -2.477 | 3.862E-08 |
| FC_GAMMA_R_MEDIATED_PHAGOCYTOSIS | -2.091 | 6.261E-07 |
| SYSTEMIC_LUPUS_ERYTHEMATOSUS | -2.207 | 1.100E-06 |
| TYPE_I_DIABETES_MELLITUS | -2.351 | 1.163E-06 |
| CHEMOKINE_SIGNALING_PATHWAY | -1.880 | 1.849E-06 |
| JAK_STAT_SIGNALING_PATHWAY | -1.966 | 1.881E-06 |
| NOD_LIKE_RECEPTOR_SIGNALING_PATHWAY | -2.139 | 5.082E-06 |
| FATTY_ACID_METABOLISM | 2.197 | 6.982E-06 |
| NEUROACTIVE_LIGAND_RECEPTOR_INTERACTION | -1.675 | 7.516E-06 |
| ALLOGRAFT_REJECTION | -2.258 | 1.031E-05 |
| INTESTINAL_IMMUNE_NETWORK_FOR_IGA_PRODUCTION | -2.189 | 1.095E-05 |
| CITRATE_CYCLE_TCA_CYCLE | 2.195 | 1.230E-05 |
| HEMATOPOIETIC_CELL_LINEAGE | -1.981 | 1.772E-05 |
| ANTIGEN_PROCESSING_AND_PRESENTATION | -1.944 | 2.551E-05 |
| GLYCINE_SERINE_AND_THREONINE_METABOLISM | 2.138 | 3.855E-05 |
| NATURAL_KILLER_CELL_MEDIATED_CYTOTOXICITY | -1.845 | 5.073E-05 |
| TOLL_LIKE_RECEPTOR_SIGNALING_PATHWAY | -1.855 | 9.737E-05 |
| VALINE_LEUCINE_AND_ISOLEUCINE_DEGRADATION | 2.047 | 1.093E-04 |
| STEROID_BIOSYNTHESIS | 2.109 | 1.149E-04 |
| PRIMARY_BILE_ACID_BIOSYNTHESIS | 2.064 | 1.343E-04 |
| AMINOACYL_TRNA_BIOSYNTHESIS | 2.036 | 5.036E-04 |
| PRION_DISEASES | -1.977 | 5.836E-04 |
| PRIMARY_IMMUNODEFICIENCY | -1.976 | 5.943E-04 |
| PATHOGENIC_ESCHERICHIA_COLI_INFECTION | -1.839 | 6.018E-04 |
| AUTOIMMUNE_THYROID_DISEASE | -1.865 | 8.764E-04 |
| LYSINE_DEGRADATION | 1.826 | 9.741E-04 |
| PPAR_SIGNALING_PATHWAY | 1.767 | 1.457E-03 |
| CELL_ADHESION_MOLECULES_CAMS | -1.568 | 1.672E-03 |
| TERPENOID_BACKBONE_BIOSYNTHESIS | 1.909 | 1.963E-03 |
| ADIPOCYTOKINE_SIGNALING_PATHWAY | 1.682 | 2.564E-03 |
| T_CELL_RECEPTOR_SIGNALING_PATHWAY | -1.560 | 2.571E-03 |
| PROPANOATE_METABOLISM | 1.821 | 2.639E-03 |
| BUTANOATE_METABOLISM | 1.813 | 2.986E-03 |
| GLYCOSYLPHOSPHATIDYLINOSITOL_GPI_ANCHOR_BIOSYNTHESIS | 1.848 | 3.100E-03 |
| PROTEIN_EXPORT | 1.851 | 3.886E-03 |
| ARGININE_AND_PROLINE_METABOLISM | 1.648 | 3.946E-03 |
| BIOSYNTHESIS_OF_UNSATURATED_FATTY_ACIDS | 1.874 | 4.181E-03 |
| VASCULAR_SMOOTH_MUSCLE_CONTRACTION | 1.462 | 5.161E-03 |
| SNARE_INTERACTIONS_IN_VESICULAR_TRANSPORT | 1.617 | 7.142E-03 |
| REGULATION_OF_ACTIN_CYTOSKELETON | -1.390 | 8.182E-03 |
| TRYPTOPHAN_METABOLISM | 1.617 | 8.441E-03 |
| ARRHYTHMOGENIC_RIGHT_VENTRICULAR_CARDIOMYOPATHY_ARVC | -1.539 | 9.051E-03 |
| ALANINE_ASPARTATE_AND_GLUTAMATE_METABOLISM | 1.617 | 1.001E-02 |
| HISTIDINE_METABOLISM | 1.643 | 1.043E-02 |
| INSULIN_SIGNALING_PATHWAY | 1.446 | 1.160E-02 |
| CARDIAC_MUSCLE_CONTRACTION | 1.518 | 1.376E-02 |
| B_CELL_RECEPTOR_SIGNALING_PATHWAY | -1.451 | 1.399E-02 |
| DRUG_METABOLISM_CYTOCHROME_P450 | 1.502 | 1.664E-02 |
| MAPK_SIGNALING_PATHWAY | -1.290 | 1.725E-02 |
| PYRUVATE_METABOLISM | 1.524 | 1.807E-02 |
| ASTHMA | -1.542 | 1.849E-02 |
| BASE_EXCISION_REPAIR | 1.544 | 2.029E-02 |
| VIRAL_MYOCARDITIS | -1.463 | 2.140E-02 |
| TGF_BETA_SIGNALING_PATHWAY | -1.469 | 2.251E-02 |
| LEUKOCYTE_TRANSENDOTHELIAL_MIGRATION | -1.386 | 2.579E-02 |
| HYPERTROPHIC_CARDIOMYOPATHY_HCM | -1.371 | 3.169E-02 |
| TYROSINE_METABOLISM | 1.442 | 3.254E-02 |
| NUCLEOTIDE_EXCISION_REPAIR | 1.472 | 3.344E-02 |
| MISMATCH_REPAIR | 1.585 | 3.480E-02 |
| MATURITY_ONSET_DIABETES_OF_THE_YOUNG | 1.545 | 3.618E-02 |
| PATHWAYS_IN_CANCER | -1.237 | 3.666E-02 |
